# Hydrogen for Heating: Technologies, Challenges, and Opportunities

**DOI:** 10.12688/openreseurope.20432.3

**Published:** 2026-01-15

**Authors:** Oleh Sokil, Nazar Podolchak, Iryna Stetsiv, Mykhailo Zuiev, Bohdan Chepil

**Affiliations:** 1 Department of Financial and Administrative Management, Lviv Polytechnic National University, Lviv, 79013, Ukraine

**Keywords:** hydrogen heating systems; green hydrogen; energy decarbonization; combined heat and power (CHP); heat pumps; hydrogen infrastructure; smart energy systems; public-private partnerships.

## Abstract

Hydrogen is gaining prominence as a key enabler in the global shift toward low-carbon energy systems, yet its role in heating—particularly in residential, commercial, and industrial contexts—remains underdeveloped. This paper explores the potential of hydrogen-based technologies to decarbonize heating, focusing on technological innovations, economic feasibility, and regulatory frameworks. Drawing on a comprehensive review of literature, policy documents, and case studies such as the EU-supported H2Heat project, the paper examines developments in hydrogen production, storage, and distribution, with a special emphasis on green hydrogen and its integration into Combined Heat and Power (CHP) systems and heat pump technologies. The findings demonstrate the quick advancements in infrastructure prepared for hydrogen, electrolyzer efficiency, and renewable energy-based hybrid energy systems. High costs, infrastructure retrofitting, safety issues, and regulatory fragmentation are still problems, though. Hydrogen heating has a lot of potential, especially for hard-to-electrify industries and seasonal storage requirements, the study concludes, but its success hinges on concerted policy action, investment incentives, and international cooperation. By providing strategic recommendations for scaling hydrogen heating solutions and establishing them as a feasible part of sustainable energy transitions, the paper adds to the current conversation.

## Introduction

Hydrogen is currently perceived as a critical energy carrier in moving towards a more sustainable and low-carbon economy for the world. It has been extensively studied due to its potential as a clean alternative to fossil fuels in different sectors, such as transportation, industrial processes, and power generation. However, applying it to heating applications-both residential and industrial-presents unique challenges and opportunities that are vital to the development of energy systems. Hydrogen is important for heating because it makes it possible to decarbonize energy consumption in buildings and industry, generally within industries that have traditionally had high carbon footprints.

Current trends reflected by such researchers Chen *et al.* (2023); Benalcazar and Komorowska (2024) are toward increased deployment of hydrogen technologies for heat, encouraged by technology development, policy support, and environmental concerns. However, significant challenges remain in the development and deployment of hydrogen heating. Key challenges include infrastructure retrofitting; the economics of hydrogen production, storage, and distribution; and the technology readiness of end-use equipment. Finally, safety risks related to the high flammability of hydrogen must be taken into account and appropriately managed.

Overcoming these challenges will require various approaches: research and development, policy and regulatory frameworks, and public-private partnership. This paper will attempt to review the state of the art in hydrogen technologies for heating, outline the main challenges which are still presenting significant barriers to wide-spread technology adoption, and set out opportunities that exist for overcoming these challenges to achieve a wider diffusion of hydrogen heating technologies.

The paper will review these elements with the intent of contributing to the ongoing discourse on the role of hydrogen in accomplishing heating decarbonization and outlining some future research and policy directions that could quicken the deployment of hydrogen-based heating solutions.

In this respect, hydrogen as a means of heating is very promising for reducing greenhouse gas emissions both in the residential and industrial sectors. Despite the promise of hydrogen for heating, there are a lot of challenges that face its adoption in scaling up and practical application.

Many existing heating infrastructures, particularly those designed for conventional natural gas use, require varying degrees of retrofitting or, in some cases, complete overhauls to accommodate hydrogen, depending on the specific system configuration and hydrogen concentration. For instance, in the H2HEAT project at Chuimi SCS, partial blending of hydrogen was implemented without a full replacement of boiler systems.

Nonetheless, the majority of conventional heating infrastructure is tailored to the physical characteristics of methane (CH _4_), not hydrogen (H _2_). Hydrogen’s lower molecular weight allows it to permeate seals more easily, increasing the risk of leaks; its higher diffusivity enhances the likelihood of leakage and explosion; and its broad flammability range imposes stricter safety requirements. Additionally, hydrogen can cause embrittlement in certain metals. Therefore, adaptation often requires replacing seals, valves, and burners; inspecting or substituting pipelines with H _2_-resistant materials; installing advanced leak detection and control systems; and retrofitting or completely replacing boilers with hydrogen-ready models. According to the UK Hydrogen Strategy, upgrading just the distribution infrastructure alone is estimated to cost several billion pounds ( BEIS, 2021).

To address the issue of hydrogen embrittlement in metallic pipelines originally designed for natural gas (CNG), emerging materials science approaches propose the use of internal polymer linings or thermal spray coatings. These barrier layers act as hydrogen diffusion inhibitors, preventing atomic hydrogen from penetrating the pipeline metal lattice, which can lead to material degradation over time. Thermally sprayed polymer coatings, particularly those based on fluoropolymers or epoxy resins, have shown promise in laboratory conditions due to their high chemical resistance and adhesion to steel substrates. Furthermore, multilayer composite solutions that combine mechanical strength with hydrogen resistance are under development to retrofit existing infrastructure without the need for full replacement ( Jansen & Reins, 2024; Melo *et al.*, 2024). These innovations may offer a cost-effective and technically feasible pathway for adapting current CNG pipeline networks to safely transport hydrogen, especially in regions where large-scale infrastructure replacement would be economically or logistically prohibitive ( Islam *et al.*, 2025).

Economic feasibility has become the second challenge to hydrogen heating systems at the moment, as long as their production-mostly via electrolysis with renewable feedstocks-costs a great deal. Hydrogen heating systems also require large storage and distribution facilities in order to achieve some kind of energy balance, given the low energy density compared with traditional fuels.

Besides that, regulatory and safety issues are also major concerns. The highly flammable nature of hydrogen demands high standards for safety and standards that are not only uniform across various regions but also still under development for wide-spread facilitation of this fuel. The regulatory framework also lags behind technological advancement, making the gap between potential pilot projects and full-scale deployment wider.

These are exacerbated by the slow market acceptance of hydrogen heating solutions due to limited consumer awareness and available alternative low-carbon heating technologies like heat pumps and biomass, which enjoy mature markets and policy support.

These are problems that require, beyond technological innovations and investments, coordination at the policy and industry levels, as well as among the research community, for practical and economically viable solutions.

The primary goal of this paper is to critically evaluate the state of affairs and future prospects of hydrogen as a significant energy vector for heating applications. To this end, it conducts a thorough analysis of the most recent technological advancements, a socio-economic and environmental issues analysis, and a strategic exploration of the opportunities that may present numerous opportunities for the spread of hydrogen-based heating systems. In particular, the main aims of paper are:

- Review technological advancements: outline the current state of the art in hydrogen production, with a focus on green hydrogen derived from renewable resources, retrofitting existing heating systems, and new technological advancements that allow for the safe and effective use of hydrogen for heating.
- Identify and analyze challenges: To look at the various technical, economic, and regulatory barriers that currently impede the integration of hydrogen heating systems. Among those are infrastructure issues, cost competitiveness, market readiness, and regulatory compliance.
- Explore opportunities for advancement: To point out possible pathways and innovative approaches to overcome existing challenges. This section will debate the role government policies will play, the use of financial incentives, public-private partnerships, and community engagement in promoting hydrogen heating solutions.

In addition to offering strategic recommendations based on review and analysis, the paper will offer suggestions for accelerating the use of hydrogen for heating for all stakeholders, including academics, industry leaders, and policymakers. The ultimate goal of this paper is to contribute to the body of knowledge that supports the shift to a more sustainable heating sector and helps shape the practices and policies that will determine how hydrogen is used in energy systems around the world in the future.

## Materials and methods

Technological developments in hydrogen heating have been widely studied, including adaptations of traditional heating systems such as hydrogen burners and boilers. Research by Marchenko and Smikhula (2023) demonstrated the viability of retrofitting existing natural gas boilers to operate on hydrogen, detailing modifications required and their impact on efficiency and emissions. The authors showed that condensing boilers offer superior performance due to their ability to recover a greater proportion of thermal energy—specifically, by condensing water vapor from flue gases generated during the combustion of methane (or natural gas). They also proposed specific boiler and burner designs suitable for hydrogen combustion and its mixtures with methane or natural gas. If pure hydrogen replaces natural gas in pipelines, the volumetric leak-age rate (m³/s) would increase by approximately 2.8 times. However, the associated energy loss would decrease by around 9.2%, reflecting the lower specific energy losses during hydrogen transport compared to methane. Green hydrogen integration into biorefineries facilitates local optimization, reducing both costs and the environmental footprint related to long-term storage and transport. The widespread availability of green hydrogen and biomass in rural settings presents unique possibilities for their synergistic use, as discussed by Giuliano *et al.* (2024).

Studies like those by Matute *et al.* (2021) evaluate the costs of producing, storing, and distributing hydrogen in comparison to traditional fuels, making economic assessments a major area of focus. These studies frequently come to the conclusion that in order to make hydrogen competitive, substantial subsidies or technological advancements are required. Market potential analyses, such as those conducted by You *et al.* (2016), offer a thorough examination of the markets' readiness for hydrogen heating technologies, highlighting important industries and geographical areas where hydrogen may be adopted early on because of advantageous regulatory and economic conditions.

A breaf review of the policies regarding the use of hydrogen (H2) for heating purposes encompass national policies in Spain, as well as broader EU and international directives. Here's an overview across these three levels:

- National (Spain). Spain has increasingly focused on integrating hydrogen into its energy mix, especially in the context of its National Hydrogen Strategy and Hydrogen Roadmap: a commitment to renewable hydrogen ( Green Hydrogen Organization, 2024). This strategy aims to develop green hydrogen production, primarily for industrial use, but it also explores the potential for hydrogen in heating applications within the residential and commercial sectors. Spain's commitment to hydrogen aligns with its broader objectives for renewable energy and decarbonization by 2050.
- European Union (EU). The EU's Hydrogen Strategy complements national plans like those of Spain. It promotes the adoption of green hydrogen, produced via electrolysis powered by renewable energy sources, as part of the bloc's transition to a carbon-neutral economy ( EU energy policy, 2024). The strategy outlines investments and regulatory frameworks intended to scale up hydrogen production, infrastructure, and market deployment. For heating, the EU considers hydrogen as a viable option particularly in sectors and regions where electrification is less feasible.
- Globally, hydrogen policies vary significantly but are gaining momentum as part of the energy transition dialogue. International bodies such as the International Energy Agency (IEA) and the International Renewable Energy Agency (IRENA) ( International Renewable Energy Agency (IRENA), 2022) advocate for the role of hydrogen in achieving global energy and climate goals. These organizations provide guidelines and frameworks that encourage member countries to explore hydrogen as part of their energy strategies, including for heating purposes. Additionally, international collaborations and partnerships, such as the Hydrogen Council, promote policy development, market creation, and technology sharing across borders ( Prysiazhniuk, 2023).

Regulatory and safety considerations are also crucial, with works like Jansen and Reins (2024) and Melo *et al.* (2024) reviewing existing safety standards and regulatory policies that impact the deployment of hydrogen heating solutions. Detailed risk assessments, such as those by Rusin *et al.* (2024), offer guidelines for safe storage and handling practices in residential and industrial settings, addressing concerns related to hydrogen's flammability.

Trends also reveal a growing focus on decentralized hydrogen production as a means to reduce reliance on large-scale infrastructure and associated transportation costs. This approach aligns with policies promoting localized renewable energy use for hydrogen production, enabling tailored solutions for specific heating needs. Additionally, there is an increasing emphasis on integrating hydrogen systems with smart energy management technologies, allowing for dynamic adjustments in production and distribution based on demand patterns and renewable energy availability.

The evolution of hydrogen policies for heating demonstrates a clear trajectory toward achieving a low-carbon energy landscape. As these frameworks mature, continued emphasis on harmonization, innovation, and public engagement will be essential in realizing hydrogen’s full potential as a sustainable heating solution.

## Results

The Nnabuife *et al.* (2023) paper provides a comprehensive analysis of various hydrogen production methods and their environmental impacts. It underscores the importance of considering environmental consequences alongside technical feasibility when assessing the effectiveness of these methods. The study clearly states that no single method emerges as superior, as each possesses distinct advantages and disadvantages concerning efficiency, scalability, cost-effectiveness, and environmental impact. Green hydrogen offers a viable solution for lowering carbon emissions in various global economic sectors. Its dual role as both an energy source and an industrial raw material, along with its capacity for emission-free production, makes it a crucial component in shifting towards a sustainable energy landscape. Green hydrogen is particularly valuable for its potential to reduce emissions in sectors that are difficult to decarbonize, including heavy industry, long-distance transportation, and applications requiring high-temperature heat, positioning it as a critical tool in broad-based climate change efforts ( Banerjee & Lnu, 2024).

The review reveals a dynamic field characterized by rapid advancements and a strong focus on overcoming the economic and technical barriers to hydrogen's viability as a heating solution. While promising, the literature consistently highlights the need for supportive policies, further technological innovation, and comprehensive safety and regulatory frameworks to fully realize the potential of hydrogen in heating applications.

Green hydrogen, produced through water electrolysis powered by renewable energy sources, is at the forefront of this transformation. The process involves splitting water into hydrogen and oxygen using electricity generated from renewable resources such as solar, wind, or hydroelectric power ( Munyaneza, 2024). This method not only provides a carbon-neutral hydrogen source but also aligns with global sustainability goals by integrating renewable energy into the heating sector.

The cost of green hydrogen production is heavily influenced by the local price of electricity from renewable sources, which varies significantly by geography. Regions with abundant solar irradiance, such as India, the Middle East, and North Africa, often benefit from lower photovoltaic electricity prices—sometimes below $20/MWh—making electrolytic hydrogen more cost-competitive ( Cortez *et al.*, 2024; Singla *et al.*, 2021). In contrast, countries located at higher latitudes, such as Canada or the Nordic states, may experience higher generation costs due to seasonal variability and reduced solar availability, though these regions can compensate with wind or hydro resources to some extent. This geographic disparity affects not only the levelized cost of hydrogen (LCOH) but also the strategic planning of hydrogen infrastructure and export opportunities. Incorporating these regional dynamics into techno-economic assessments is crucial for identifying viable deployment zones and for policy frameworks that aim to ensure competitive and equitable hydrogen markets globally.

The methodologies for integrating hydrogen into existing heating infrastructures are complex and varied. Several pilot projects across Europe and Asia have demonstrated the feasibility of converting existing natural gas networks to hydrogen. These projects typically involve a phased approach, starting with a blend of natural gas and hydrogen, which gradually transitions to 100% hydrogen. The technical considerations for such transitions include assessing the material compatibility of existing pipelines, upgrading seals and fittings to handle hydrogen's unique properties, and implementing advanced monitoring systems to ensure safety and efficiency ( Ozturk *et al.*, 2023).

Storage and delivery systems are another critical area of focus. Due to hydrogen's low density, innovative solutions for efficient storage and transportation are crucial. Technologies such as liquid organic hydrogen carriers (LOHCs), metal hydrides, and advanced compression techniques are under development to address these challenges. Each method offers distinct advantages and limitations in terms of energy density, safety, and cost, influencing their suitability for different scales of operation and geographic conditions ( Moradi & Groth, 2019; Reynolds *et al.*, 2024).

The cost and performance characteristics of compressed hydrogen storage tanks vary significantly depending on the tank type and the pressure they are designed to withstand. The five primary categories—Type I through Type V—differ in both materials and structural design. Type I tanks are made entirely of metal (typically steel or aluminum) and are the least expensive but also the heaviest, limiting their use primarily to stationary applications. Type II tanks use a metal liner with partial composite wrapping, offering moderate weight reduction at a slightly higher cost. Type III tanks feature a full composite wrap over a metal liner, providing a good balance between weight and strength, making them suitable for mobile applications. Type IV tanks are fully composite with a polymer liner, enabling very high pressure (up to 700 bar) while being significantly lighter—ideal for transportation but also much more expensive. Type V tanks, still under development and not widely commercialized, aim to be linerless all-composite structures, offering the lowest weight but at very high cost and with ongoing challenges in safety certification and durability ( Muthukumar *et al.*, 2023; Wang *et al.*, 2021).

Understanding the trade-offs between cost, weight, safety, and pressure capability is crucial when selecting storage solutions for different hydrogen heating system configurations, particularly in scenarios where mobility, space, or infrastructure limitations exist.

The storage and technical challenges involved in supplying hydrogen to combined heat and power (CHP) systems for heating are complex, primarily due to hydrogen's inherent physical properties and the operational demands of CHP systems. Hydrogen has a very low density, necessitating its compression into high-pressure tanks to achieve an energy density practical for use. These tanks must be constructed from materials capable of handling high pressures while resisting hydrogen embrittlement, which can make metals brittle over time.

In addition to the type-specific storage characteristics, it is essential to consider the energy expenditure involved in each storage method. Compressing hydrogen gas for high-pressure storage requires substantial energy input, especially when targeting pressures of 350–700 bar, which are common in industrial and transportation applications. Similarly, storing hydrogen in liquid form at cryogenic temperatures (−253°C) demands significant energy for liquefaction—estimated to consume approximately 30–40% of the energy content of the hydrogen itself—along with continuous energy input to maintain such low temperatures. In contrast, solid-state hydrogen storage systems, such as metal hydrides and physisorption-based materials, typically require much less energy for maintaining storage conditions. These systems operate at moderate temperatures and pressures and may, in some cases, offer nearly passive storage with only minimal energy input needed for hydrogen release. Although solid-state options face challenges related to slow kinetics and material costs, their energy efficiency during storage makes them particularly promising for stationary applications where volume and weight constraints are less critical ( Shin & Ha, 2023; Singla *et al.*, 2021; Yadav *et al.*, 2022).

The scalability of hydrogen storage is also critical, as CHP systems vary in size and may serve large facilities, requiring substantial hydrogen volumes. Efficient compression and decompression technologies are vital to maximize energy efficiency and minimize losses, ensuring operational cost-effectiveness. Moreover, CHP systems generally require high-purity hydrogen to maintain efficiency and prevent system damage, posing additional challenges in maintaining purity, especially after long storage periods.

Safety is another significant concern, given hydrogen's high flammability. Storage systems must be designed to prevent leaks and withstand accidents, incorporating robust safety protocols and detection systems. Innovative storage solutions, such as liquid organic hydrogen carriers (LOHCs) and metal hydrides, are under development to enhance hydrogen storage. LOHCs can store hydrogen at lower pressures and ambient temperatures, although they require complex chemical processes to release hydrogen. Metal hydrides offer high-density storage but face issues with slow kinetics and thermal management.

Lastly, the cost of developing and implementing these advanced storage technologies must be considered, balancing initial setup and operational costs with the benefits of hydrogen use in CHP systems. Integrating hydrogen storage and delivery systems with existing CHP infrastructure requires careful planning and modifications, tailoring solutions to accommodate hydrogen's unique characteristics. Addressing these multifaceted storage and technical challenges is crucial for the effective and efficient deployment of hydrogen in heating applications through CHP systems.

Furthermore, the integration of hydrogen heating systems with smart energy management technologies is being explored to optimize energy use and enhance system responsiveness to market and environmental conditions. These smart systems use real-time data analytics to adjust hydrogen production and distribution based on immediate energy demand, weather conditions, and electricity prices from renewable sources.

To provide a comprehensive understanding of these methodologies, this section reviews several key studies and real-world applications that highlight the practical challenges and innovative solutions in the deployment of hydrogen heating systems. The discussion not only showcases current capabilities and limitations but also provides insight into future directions for research and development in hydrogen technologies.

### CHP and heat pump

***Other technologies.*** The exploration of technological advancements in hydrogen for heating reveals significant progress and some promising trends. Recent advancements in hydrogen production technologies, especially in electrolysis, have significantly enhanced efficiencies and reduced costs. These improvements are largely due to innovations in catalyst materials and electrolyzer designs, facilitating the broader adoption of green hydrogen production. Notably, the development of proton exchange membrane (PEM) electrolyzers has marked a considerable technological advancement. These electrolyzers operate at higher efficiencies and are capable of adjusting dynamically to the variable supply of renewable electricity, making them particularly suitable for applications such as hydrogen for heat. This technology's relevance to our focus on hydrogen for heating applications warrants a detailed review and discussion ( Nikzad *et al.*, 2024; Zeng *et al.*, 2024).

In terms of heating technologies, the adaptation of existing heating systems to utilize hydrogen has progressed notably. Recent pilot projects in regions like Northern Netherlands Hydrogen Valley (socalled HEAVENN project) have successfully demonstrated that existing natural gas networks and boilers can be converted to use hydrogen with minimal modifications. These conversions involve replacing specific components such as burners and nozzles to accommodate hydrogen’s different combustion properties, and the results so far have shown that these systems can achieve comparable heating efficiencies and reliability ( Squintani & Schouten, 2024).

Storage technologies have also seen considerable advancements. The development of solid-state hydrogen storage methods, such as metal hydrides and complex hydrides, offers safer and more efficient ways to store and release hydrogen compared to traditional high-pressure tanks. These technologies reduce the risks associated with hydrogen storage and allow for more compact and scalable solutions suitable for both residential and industrial applications ( Adaikalam *et al.*, 2024; Zeng *et al.*, 2022).

Moreover, the integration of hydrogen heating systems with smart grid technologies has begun to take shape. These systems leverage IoT sensors and real-time data analytics to optimize hydrogen production, storage, and distribution, ensuring maximum efficiency and minimal wastage. This integration not only enhances the operational efficiency of hydrogen heating systems but also contributes to broader energy system stability by providing demand-response capabilities that can balance intermittent renewable energy sources ( Agi *et al.*, 2024; Loukriz *et al.*, 2024).

In summary, the technological advances in hydrogen for heating are substantial, addressing many of the limitations that have historically hindered the deployment of hydrogen as a mainstream energy carrier. However, continuous innovation and improvement are needed to overcome remaining challenges related to cost, scalability, and system integration.

The assessment of technological advances in the use of hydrogen, referred to as hydroxy, for heating purposes involves a comprehensive evaluation of recent innovations and persistent challenges. Hydrogen offers a promising pathway for decarbonizing the heating sector, traditionally reliant on fossil fuels, through significant strides in production, storage, and utilization technologies ( Rincon *et al.*, 2024).

In the realm of hydrogen production, the efficiency and sustainability of electrolysis, a process that splits water into hydrogen and oxygen using electricity, are pivotal. Innovations in this area, particularly in electrolyzer technology such as alkaline and proton exchange membrane (PEM) systems, have reduced energy consumption and operational costs. These advances have been driven by breakthroughs in catalyst materials and membrane technologies, making green hydrogen, produced using renewable energy sources like solar or wind, a more competitive option for heating applications ( Gado *et al.*, 2024).

Storage solutions for hydrogen also represent a critical area of technological development. The challenges posed by hydrogen's low density and high flammability have led to innovative storage methods, including metal hydrides and liquid organic hydrogen carriers (LOHCs) ( Sung *et al.*, 2025). These technologies allow for safer and more efficient hydrogen storage at higher densities than conventional high-pressure tanks, addressing safety concerns and enhancing the practicality of hydrogen for both residential and commercial heating systems.

The integration of hydrogen into existing heating infrastructure has seen considerable advancement through pilot projects and the development of new technologies. Innovations such as hydrogen-ready boilers and burners, which can operate on pure hydrogen or a hydrogen-natural gas blend, facilitate the retrofitting of existing systems. This gradual integration allows for the use of hydrogen as the infrastructure and supply chains evolve, as demonstrated in projects across the Northern Netherlands, where municipal heating systems have been successfully converted to hydrogen ( Broersma *et al.*, 2024).

Furthermore, alongside technological advancements, improvements in regulatory standards and safety protocols are essential to support the safe deployment of hydrogen heating systems. These enhancements address public and regulatory concerns by incorporating advanced sensor and detection systems into installations, ensuring safe operation.

Despite these promising developments, challenges remain that must be addressed to fully leverage hydrogen's potential in heating. The high initial costs associated with establishing hydrogen production facilities and retrofitting infrastructure, along with the efficiency losses in energy conversion, continue to be significant barriers. These issues underscore the need for ongoing research and development to improve the efficiency, safety, and cost-effectiveness of hydrogen technologies.

The dynamic field of hydrogen technology for heating is characterized by rapid innovations aimed at overcoming technical and economic barriers. As these technologies continue to evolve and become more cost-effective, hydrogen is increasingly positioned as a key component in achieving a sustainable and low-carbon heating future. This ongoing transition is supported by a blend of technological advancements, regulatory enhancements, and pilot projects that collectively demonstrate hydrogen's viability as a transformative energy solution for heating.

***H2heat project.*** In the context of exploring the latest technologies for utilizing hydrogen (H2) for heating purposes, it's essential to focus on developments that go beyond the production aspect, such as electrolysis, and look more into how hydrogen is applied in heating systems. Key technologies that are shaping the use of hydrogen for heating include Combined Heat and Power (CHP) systems, the integration of CHP with heat pumps, and innovations in hydrogen transport such as tube trailer modifications ( Ge *et al.*, 2023).

Combined Heat and Power (CHP) Systems: CHP systems, also known as cogeneration systems, generate electricity and capture the heat that would otherwise be wasted to provide useful thermal energy. When these systems are powered by hydrogen, they offer a highly efficient use of fuel, which can significantly reduce greenhouse gas emissions compared to conventional fossil fuel systems. The efficiency of hydrogen CHP systems is particularly notable because they can achieve energy efficiency rates of over 80%, compared to traditional power plants which often have efficiencies around 50%. This makes hydrogen CHP a compelling option for district heating systems, industrial applications, and residential heating, especially in regions with high demands for both heating and power ( Ge *et al.*, 2023).

CHP combined with heat Pumps – main innovation of the H2Heat project. A significant innovation in the H2Heat project is the integration of CHP systems with heat pumps. This hybrid approach leverages the strengths of both technologies: the CHP system efficiently generates electricity and provides heat, while the heat pump can use a fraction of the electricity generated to extract heat from the external environment, thereby enhancing the overall efficiency of the system. This combination allows for greater flexibility and efficiency in temperature control and energy use, making it particularly effective in climates with variable heating needs. The integration also aims to maximize the utilization of green hydrogen, aligning with sustainability goals by reducing reliance on grid electricity and lowering carbon footprints.

Tube trailer modifications for hydrogen transport. The transport of hydrogen to various points of use, such as residential areas or industrial sites, is crucial for the widespread adoption of hydrogen heating technologies. Tube trailers, which are high-pressure tanks mounted on trailers, have traditionally been used to transport compressed gases. Recent modifications in these trailers include advancements in materials and engineering to safely and efficiently transport hydrogen. Innovations such as the use of lightweight composite materials help increase the payload and reduce transportation costs ( Suvar *et al.*, 2024). Moreover, advancements in tank design to handle higher pressures enable these trailers to carry larger quantities of hydrogen, improving the economics of hydrogen distribution and making it more accessible for heating applications.

The use of hydrogen for heating is advancing through innovative technologies that enhance energy efficiency and sustainability. The integration of CHP systems with heat pumps represents a forward-thinking approach to leveraging hydrogen's potential in heating, providing a model for future developments. Meanwhile, improvements in the infrastructure for hydrogen transport, like tube trailer modifications, play a crucial role in addressing logistical challenges, making hydrogen a more viable and attractive option for heating needs across various sectors. These technologies not only promise to improve the efficiency and sustainability of heating systems but also contribute to broader efforts to decarbonize energy systems globally.

**Figure 1.**
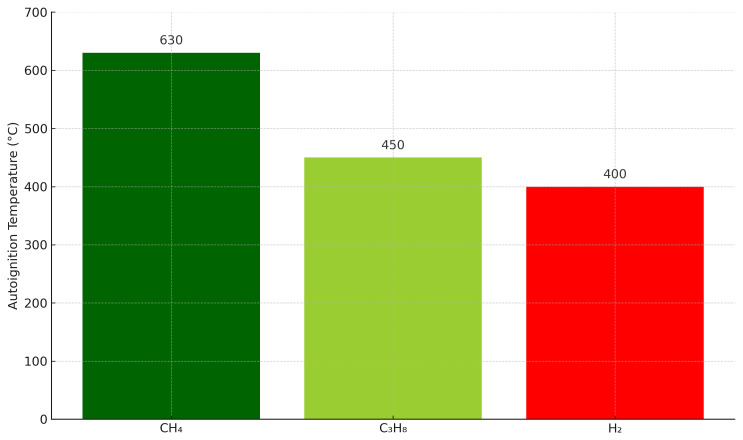
Comparison of autoignition temperatures for hydrogen (H _2_), methane (CH _4_), and propane (C _3_H _8_).

One of the main characteristics that differentiate H2 from CH4 it is its minimum auto-detonation temperature, which is the temperature above which the gas can be ignite without any external influence. In the following picture you can appreciate the auto-detonation temperature for each kind of gas ( Figure 1):

This figure highlights the lower autoignition temperature of hydrogen, which impacts combustion safety protocols in hydrogen heating systems. Within a combustion engine, the different basic consecutive stages, through which the gas passes before it reaches the combustion chamber of an engine, are : Airgas mixer, turbocharger, throttle valve and combustion chamber. As there is an increase of temperature in those stages, the probability of having ignition, and thus misusing the energy of H2 and having material damages is high. Therefore, the conventional way to prepare the gas for the engine with a conventional CHP only allows 10% of H2 mixed with CH4.

2G has been working on special materials achieving that the Agenitor 2G engine allows until 30–40% (depending on the quality of the natural gas) of H2 through the conventional way.

For 100% of H2, without any mixture, 2G designed special injectors which allow the mixture H2-air in the combustion chamber and thus uses the maximum energy from the gas.

For the H2Heat project 2G proposes the Agenitor404 cogeneration unit. The unit needs 101,8m3/h H2 to produce 115Kw electric and 129KW thermal power.

2G proposes an adapted double Heat Pump system to create a high water temperature heater of up to 82°C. It is not possible to reach this temperature with a single air-to-water heat pump. The first, air-to-water heat pump, raises the water temperature from 30°C to 35°C. The second, water-to-water Heat Pump, will use this water at 35°C as a heat source to raise the water temperature to 82°C.

Combined Heat and Power units (CHP) have been conceived to use all the energy of the engine, which produces electricity through the generator, and all the hotness produced during the process in the engine and in the exhaust gas, is harnessed in form of heat. Thus, almost all the energy contained in the gas is used in form of useful energy.

On the other hand, the heat pump produces heat energy out of electricity, with the special feature of increasing the thermal output compared to electricity. This means that with a heat pump we obtain a thermal power output that is twice or more the electrical power input.

In an application like an hospital, where heat energy is most demanded, the combination of both technologies allows the production of heat in the most efficient way. Thus with an engine of 115KW electric and 129KW thermic (total power 244KW), we will achieve 350KW heat power using hydrogen as fuel.

We present a renewable high efficiency energy production, which can be feed with the surplus energy from the renewable production of a wind power plant.

***Identify and analyze challenges.*** Despite the promising technological advances, several challenges remain that impede the broader adoption of hydrogen as a heating solution. One of the primary challenges is the cost associated with the production of green hydrogen. Although technological improvements have reduced costs to some extent, the production of hydrogen through electrolysis remains expensive due to high energy requirements and the cost of electricity from renewable sources. This is compounded by the need for substantial investments in infrastructure to support large-scale hydrogen production and distribution.

Regulatory barriers further complicate the deployment of hydrogen technologies. The lack of harmonized standards and regulations across different regions creates uncertainty for investors and operators. Without clear and consistent regulatory frame-works, the risk associated with large-scale deployment of hydrogen technologies remains high, discouraging investment and innovation in this area.

Detealy expanding on the challenges and barriers to the deployment of hydrogen technologies for heating, there are several factors to consider that complicate its adoption compared to direct use of renewable electricity and other energy sources:

- Cost and efficiency of direct heating with renewable electricity. Directly using renewable electricity for heating, such as through electric resistance heaters or heat pumps, can be cheaper and more efficient in certain contexts. Heat pumps, in particular, offer high efficiency by transferring heat rather than generating it, often resulting in lower operational costs compared to producing hydrogen through electrolysis and then using it for heating.
- Hydrogen conversion and heat production efficiency. Hydrogen's role as an energy carrier involves conversion losses. Electrolysis itself is only about 60–80% efficient, and additional energy is lost when hydrogen is converted back to electricity or heat, making it less efficient compared to direct heating methods or heat pumps powered directly by renewable energy.
- Safety concerns. Hydrogen poses significant safety challenges due to its high flammability and smaller molecular size, which can lead to leaks. For combined heat and power (CHP) systems that use hydrogen, these risks necessitate robust safety measures, complicating deployment and increasing costs.
- Standards and regulations. The deployment of hydrogen technologies requires high safety and operational standards. The lack of harmonized standards and specific regulations for hydrogen use in different regions creates barriers for consistent deployment and increases the investment risk.
- Noise issues. Both electrolysis equipment and CHP systems can generate significant noise, which may require additional mitigation measures to be acceptable in residential or commercial areas.
- Regulatory and permitting hurdles. National and local regulations and the permitting process for new technologies like hydrogen installations can be cumbersome and slow, posing significant barriers to rapid deployment.
- Public opposition. There can be substantial public resistance to new technologies, especially those involving flammable or hazardous substances like hydrogen. Public concerns about safety, environmental impacts, or changes to local landscapes can delay or block projects.
- Life cycle carbon emissions. Assessing the full life cycle carbon emissions of hydrogen production, especially if not all electricity used is sourced from renewables, is crucial. It's necessary to evaluate whether using hydrogen for heating results in a net reduction of greenhouse gases compared to other heating technologies.
- Comparison with other carriers like methanol. Methanol can also be used as an energy carrier and has some advantages over hydrogen, such as being liquid at room temperature, which simplifies storage and transport. Evaluating whether hydrogen or methanol (or another carrier) is the best option for heat production requires a comprehensive analysis of efficiency, safety, infrastructure, and environmental impact.

The environmental impact of hydrogen production, particularly blue hydrogen, which involves carbon capture and storage (CCS), is a subject of ongoing debate ( Faber *et al.*, 2025; Lan & Yao, 2022). While blue hydrogen is seen as a stepping stone towards a hydrogen economy ( Idris-Idah *et al.*, 2024), concerns about the efficiency of CCS technologies and the ultimate fate of captured carbon highlight the need for stringent environmental assessments and robust regulatory oversight.

These challenges underscore the need for a coordinated approach that includes technological innovation, regulatory reform, and public engagement to realize the full potential of hydrogen heating systems. Addressing these issues will require collaborative efforts from government bodies, industry stakeholders, and the research community.

***Explore Opportunities for Advancement.*** The challenges identified present significant opportunities for advancement in the field of hydrogen heating. The increasing focus on decarbonization globally provides a strong impetus for the development and deployment of hydrogen technologies. Governments and international agencies can play a pivotal role by providing funding for research and development and by establishing incentives for the adoption of hydrogen technologies, such as subsidies, tax breaks, and grants.

There is also an opportunity to leverage advancements in renewable energy technologies to reduce the cost of green hydrogen production. As the cost of solar and wind energy continues to decline, coupling these energy sources directly with electrolysis can make hydrogen production more economically viable. Moreover, the development of decentralized hydrogen production facilities can help reduce transportation costs and energy losses, making hydrogen more accessible for local heating applications ( Cortez *et al.*, 2024).

National-international partnerships can be instrumental in accelerating the development of hydrogen infrastructure. By sharing risks and resources, these partnerships can tackle large-scale projects that might not be feasible for individual entities. Such collaborations can also foster innovation through the exchange of knowledge and expertise between the domestic and international experience ( Sokil *et al.*, 2022; Zbarsky *et al.*, 2020).

The H2HEAT project is an example, supported by the EU's Horizon program during 2023–2028, exemplifies a strategic initiative to harness the potential of hydrogen in transforming heating solutions within the commercial sector. This project demonstrates the complete value chain of green hydrogen production from renewable energy and its utilization in commercial building heating, particularly within hospital facilities in the Canary Islands.

The H2HEAT project features a robust consortium of participants, each contributing unique expertise to ensure the project's success:

- Platform Oceanic of the Canary Islands (PLOCAN) - Coordinates the project, leveraging its maritime research facilities to support hydrogen production.
- Esteyco S.A (EST) - Provides engineering expertise and integrates offshore renewable energy solutions.
- Neodyne Ltd - Focuses on the advanced energy management systems essential for integrating hydrogen production with renewable energy sources.
- Stargate Hydrogen Solutions OÜ - Specializes in the development of innovative electrolysis technologies, crucial for efficient hydrogen production.
- 2-G - Implements hydrogen-powered cogeneration systems that significantly enhance heating efficiency.
- International Consortium of Research Staff Associations Ltd (ICoRSA) - Contributes to policy development and research dissemination.
- Cluster Maritime Canaria (CMC) - Supports with maritime and logistical expertise, fostering regional development.
- Canary Islands Health Service (SCS) - Acts as an end-user, applying hydrogen solutions to decarbonize hospital heating systems.
- European Marine Energy Centre Ltd (EMEC) - Provides expertise in marine energy and its application in hydrogen production.
- Government of the Canary Islands (GobCan) - Supports regulatory and policy frameworks to facilitate project implementation.
- Scientific Park Lviv Polytechnic National University (SPLP) - Engages in research and development, particularly in materials and technology innovations for hydrogen applications.

**Figure 2.**
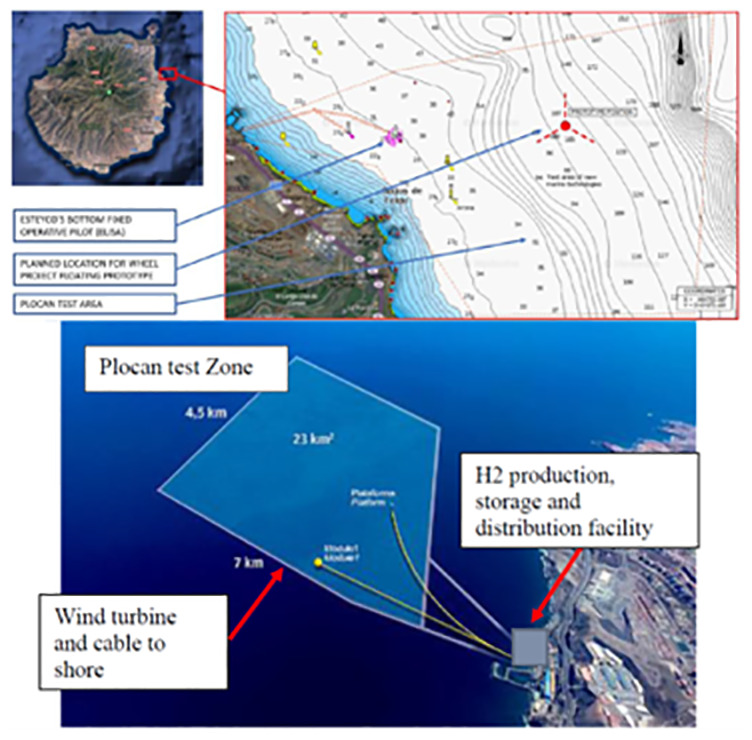
Schematic layout of the H2HEAT hydrogen production facility near Las Palmas, Canary Islands.

The H2HEAT project aims to establish a replicable model for hydrogen production and utilization that could be extended across the Canary Islands and beyond. A schematic layout of hydrogen production facilities is presented in Figure 2.

This facility uses offshore wind energy to produce green hydrogen via electrolysis, which is then used for heating applications. The figure shows the integration of renewable energy generation and hydrogen production infrastructure. The H2Heat facility near Las Palmas includes:

- Innovative Production: Utilizing a 0.5MWh electrolyser developed by Stargate, the project will produce high-purity hydrogen and oxygen, leveraging renewable energy from Esteyco's turbines.
- Advanced Cogeneration Technology: 2G's hydrogen-powered cogeneration technology will be employed to provide efficient heating solutions, demonstrating major advances in technology and integration.
- Comprehensive Digital Integration: Featuring sophisticated control systems including energy management (EMS), demand management (DMS), and SCADA to optimize the hydrogen production and usage processes.
- Environmental and Economic Benefits: The project supports the Canary Islands' 'Health Zer0 net Emissions Strategy 2030', aiming for deep decarbonization in the health sector and contributing to the broader EU goal of reducing greenhouse gas emissions.

This project not only addresses the technical feasibility of hydrogen in heating but also explores socio-economic impacts, regulatory challenges, and environmental benefits, paving the way for wider adoption and integration of hydrogen technologies in Europe and globally.

The present issues and conclusions already made stemming from the H2HEAT project underscore the necessity for robust planning and collaborative efforts to fully leverage the benefits of hydrogen technologies. To support the scalable and sustainable deployment of hydrogen-based heating technologies, the following strategic recommendations are proposed:

- integrate hydrogen heating into broader national and international energy strategies, ensuring alignment with long-term decarbonization goals and sectoral interdependencies across electricity, gas, and heating networks;
- establish harmonized safety protocols and regulatory standards to reduce operational risks and create a coherent legal environment that supports hydrogen deployment across different jurisdictions;
- develop clear and practical guidelines for retrofitting existing infrastructure and constructing new hydrogen-compatible systems, addressing technical aspects such as material compatibility, system conversion, and safety monitoring;
- promote targeted educational and public outreach initiatives to enhance public awareness, trust, and acceptance of hydrogen technologies, particularly in regions undergoing energy transitions;
- increase public and private investment in research and development, prioritizing innovation in electrolyzer efficiency, hydrogen storage, and integrated heating system design to improve performance and reduce levelized costs;
- encourage public-private partnerships and cross-sectoral collaboration to share risks, mobilize funding, and accelerate commercialization of hydrogen heating solutions;
- support international cooperation and knowledge exchange platforms, enabling pilot project replication, standard-setting, and rapid diffusion of best practices across regions.

**Figure 3.**
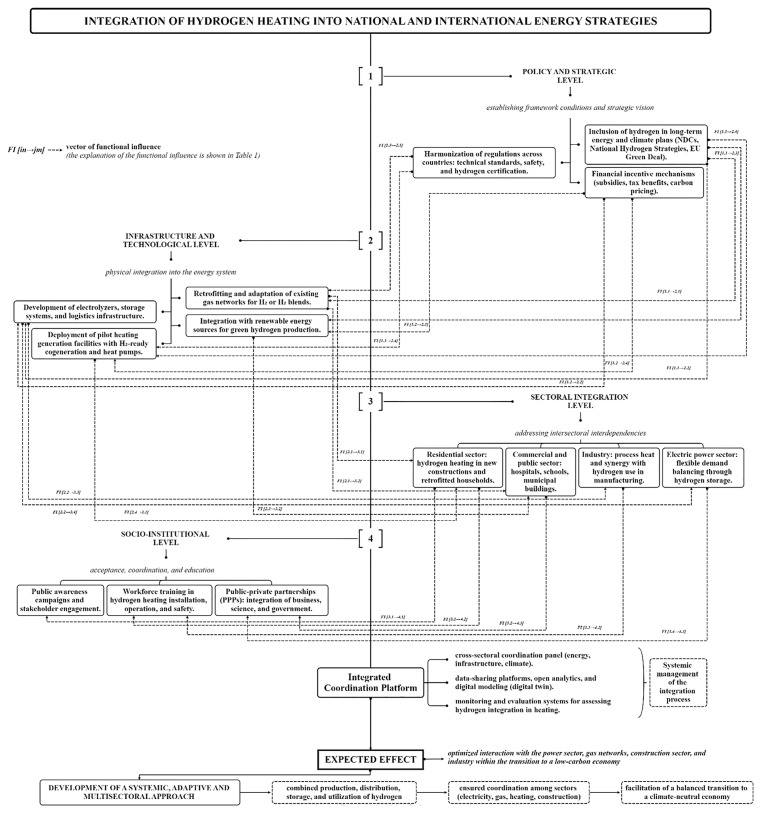
Strategic integration of hydrogen heating in national and international energy plans.

These recommendations aim to build an enabling ecosystem that facilitates the safe, efficient, and equitable integration of hydrogen into the global heating landscape. The implementation of the proposed measures requires a comprehensive strategy that integrates hydrogen heating into broader national and international energy plans, and takes into account the interdependencies between different energy systems and sectors to optimize the overall energy landscape and facilitate the transition to a low-carbon economy. An example of how such a strategy could be structured is shown in Figure 3.

This figure illustrates the interdependencies between policy frameworks, infrastructure development, and technological advancements necessary for large-scale hydrogen adoption in heating applications. The strategic inclusion of hydrogen in long-term climate and energy policies directly supports infrastructure development by establishing a clear investment trajectory. Regulatory harmonization ensures that technical and safety requirements for hydrogen systems are standardized, enabling safe retrofitting of existing gas networks and large-scale deployment of hydrogen technologies. Moreover, financial mechanisms such as subsidies and carbon pricing create the necessary market conditions for accelerating capital-intensive projects, including electrolyzer installation, hydrogen storage, and the integration of renewable energy sources for green hydrogen production.

Comparative Analysis of Hydrogen Technologies for Heating: Boilers, CHP Units, PEM Fuel Cells, and Heat Pumps

In this section, we aim to provide a clear comparison between the core hydrogen technologies discussed in this paper—hydrogen boilers, Combined Heat and Power (CHP) units, Proton Exchange Membrane (PEM) fuel cells, and heat pumps. Each technology operates under different conditions, with varying costs, efficiencies, and readiness for deployment ( Table 1).

**Table 1.** Comparative Analysis of Hydrogen Technologies for Heating.

Technology	Efficiency (thermal)	Efficiency (electrical)	Typical Operating Temperature	Cost Drivers	Technological Readiness	Use Case
**Hydrogen Boilers**	85–95%	N/A	40–80°C	Installation, retrofitting, burner technology	Short-term	Residential & industrial heating with hydrogen or methane blends
**CHP Units (Hydrogen)**	80–85%	30–40%	70–100°C	Infrastructure, hydrogen storage, cost of integration	Short-term	Large-scale industrial and district heating
**PEM Fuel Cells**	N/A	40–60%	50–90°C	Fuel purity, electrolyzer cost, system complexity	Long-term	Niche applications, off-grid, low-emission zones
**Heat Pumps**	300–400%	N/A	30–60°C (air-to-water)	Initial cost of heat pump, electricity cost	Short-term	Residential heating, district systems with direct renewable electricity

Short-Term vs. Long-Term Deployment

1. Hydrogen Boilers: These systems are relatively mature and offer the quickest pathway to hydrogen adoption for heating. They are well-suited for retrofitting existing natural gas boilers and offer efficiency rates between 85–95%. These systems can be deployed in residential and small industrial settings with a focus on blending hydrogen with natural gas as a transitional solution. Given the current infrastructure and technical readiness, hydrogen boilers are considered short-term deployable.

2. CHP Units (Hydrogen): CHP units that use hydrogen can achieve both electrical and thermal efficiencies. These systems are particularly well-suited for industrial applications and large district heating systems where both power and heat are required. However, the cost of upgrading existing infrastructure to support 100% hydrogen or hydrogen-natural gas blends remains a significant barrier, making CHP units a short-term technology for regions with existing natural gas networks. They offer high operational efficiency and lower emissions, making them ideal for large-scale industrial use.

3. PEM Fuel Cells: PEM fuel cells convert hydrogen to electricity with high efficiency (40–60%) and are considered a long-term solution for specific applications, particularly off-grid or urban environments. They are ideal for small-scale, high-efficiency applications, but their high capital cost, need for hydrogen purity, and complex integration make them less suitable for large-scale heating projects at this stage. PEM fuel cells are best suited for long-term deployment, particularly as the technology matures and hydrogen production becomes cheaper.

Heat Pumps: Heat pumps, particularly when powered by renewable electricity, provide extremely high efficiency (300–400%) by transferring heat rather than generating it. They are currently the most cost-effective and energy-efficient solution for many heating applications, particularly in residential and small industrial systems. Since they rely on electricity, their deployment is more closely tied to the availability of low-carbon grid power or on-site renewable generation. Heat pumps are short-term deployable for residential heating and as part of district heating solutions.

1. Numerical Cost Comparisons

• €/kg H _2_: Provide a clearer numerical comparison of the cost of hydrogen production, specifically focusing on green hydrogen produced through electrolysis. For instance, as of recent estimates, the cost of producing green hydrogen ranges from €3 to €7 per kg in regions with low-cost renewable electricity (e.g., wind-rich areas). This price may decrease over the next decade as electrolyzer efficiency improves and economies of scale are achieved.

• CAPEX of Electrolyzers: The capital expenditures (CAPEX) for large-scale electrolyzers are a critical factor in the economics of hydrogen production. Presently, the cost of electrolyzers is around €800-€1,500 per kW of capacity. However, it is projected that the cost will fall by 30–40% by 2030 as technology scales and innovation in materials and manufacturing techniques progress. This will significantly reduce the cost of hydrogen production and improve its competitiveness against other energy carriers.

• Cost of Retrofitting Gas Grids: Retrofitting existing gas networks to accommodate hydrogen is an expensive but necessary step for large-scale hydrogen adoption. For example, estimates indicate that the cost to upgrade natural gas pipelines to hydrogen-ready infrastructure ranges between €2 billion to €8 billion per 1000 km, depending on the region and pipeline materials. However, this cost can be reduced through the use of advanced coatings, selective retrofitting, and hybrid gas blending approaches.

2. Regional Cost Variability Tied to Renewable Electricity Prices

• Electricity Prices and Regional Variability: The cost of green hydrogen is closely linked to the price of renewable electricity. For example ( International Energy Agency (IEA), 2023):

° In regions with abundant solar or wind resources, such as the Middle East, North Africa, and parts of Southern Europe, the cost of electricity can be as low as €20-€30 per MWh, enabling hydrogen production costs of €2-€4 per kg.

° In high-latitude regions, such as Scandinavia or Canada, renewable electricity costs may be higher, closer to €40-€60 per MWh, leading to hydrogen production costs of €5-€7 per kg. These regional differences impact the economic viability of hydrogen and should be factored into policy and investment decisions.

3. Centralized vs. Decentralized Hydrogen Production Economics

• Centralized Hydrogen Production: Centralized production involves large-scale electrolyzers located near renewable energy sources (e.g., wind farms, solar fields). The economies of scale associated with centralized production can drive down the cost of hydrogen significantly, making this approach ideal for large-scale industrial applications or export. However, the cost of building hydrogen transport infrastructure (pipelines, tankers) can be significant and add to the overall cost structure.

Decentralized Hydrogen Production: Decentralized production refers to smaller, more localized electrolyzers that produce hydrogen on-site, often powered by local renewable resources. While the CAPEX per unit of installed electrolyzer capacity is higher compared to centralized systems, this model reduces transportation costs and can be advantageous for local heating applications or off-grid areas. Decentralized production is well-suited for residential or smaller-scale industrial heating needs but may face higher per-unit hydrogen costs due to the lack of economies of scale.

## Discussion

Each policy measure directly supports or enables specific infrastructure and technological actions. The effective integration of hydrogen heating depends on the synergy between strategic frameworks and physical implementation mechanisms. Only through coordinated interaction between these levels can scalable, safe, and economically viable hydrogen-based heating solutions be realized. For example, each infrastructure measure at Level 2 enables or accelerates hydrogen adoption in specific end-use sectors at Level 3. These interconnections reflect the practical pathways through which technical readiness translates into operational deployment, making the infrastructure-technological level a foundational enabler of sectoral integration. Sector-specific applications of hydrogen heating (Level 3) create direct demand for institutional support mechanisms (Level 4). Public trust, qualified labor, and coordinated partnerships are critical to the long-term viability of hydrogen integration across buildings, industry, and energy systems. Thus, socio-institutional frameworks are not merely complementary — they are structurally necessary for sustaining sectoral transformation ( Table 2).

**Table 2.** The key functional link between the levels of integration of hydrogen heating in national and international energy strategies *.

Level of integration of hydrogen heating in national and international energy strategies		Influence FI [in→jm]	Explanation of functional influence
1.1. Inclusion of hydrogen in long-term energy and climate plans	2.1. Retrofitting and adaptation of existing gas networks for H _2_ or H _2_ blends	FI [1.1→2.1]	Provides strategic support for network modernization and hydrogen deployment expansion
	2.2. Development of electrolyzers, storage systems, and logistics infrastructure	FI [1.1→2.2]	Guides national demand and investment focus on key elements of hydrogen infrastructure
	2.3. Integration with renewable energy sources for green hydrogen production	FI [1.1→2.3]	Aligns hydrogen and renewable energy strategies through common policy goals
	2.4. Deployment of pilot heating generation facilities	FI [1.1→2.4]	Demonstrates political will for innovation and supports demonstration projects
1.2. Harmonization of regulations across countries	2.1. Retrofitting and adaptation of gas networks	FI [1.2→2.1]	Enables cross-border implementation and simplifies permitting and approval processes
	2.4. Deployment of pilot heating generation facilities with H _2_-ready technologies	FI [1.2→2.4]	Standardizes safety, certification, and technical requirements
1.3. Financial incentive mechanisms (subsidies, tax benefits, carbon pricing)	2.2. Development of electrolyzers, storage systems, and logistics	FI [1.3→2.2]	Makes projects economically viable and attractive for investors
	2.3. Deployment of pilot heating generation facilities	FI [1.3→2.3)	Lowers market entry barriers and supports technology scaling
	2.4. Integration with renewable energy sources	FI [1.3→2.4]	Increases the competitiveness of green hydrogen compared to fossil-based options
2.1. Retrofitting and adaptation of existing gas networks for H _2_ or H _2_ blends	3.1. Residential sector: hydrogen heating in new constructions and retrofitted households	FI [2.1→3.1]	Enables low-disruption deployment of hydrogen heating through existing distribution networks
	3.2. Commercial and public sector: hospitals, schools, municipal buildings	FI [2.1→3.2]	Facilitates access to hydrogen fuel in public buildings with minimal infrastructure replacement
2.2. Development of electrolyzers, storage systems, and logistics infrastructure	3.3. Industry: process heat and synergy with hydrogen use in manufacturing	FI [2.2→3.3]	Provides stable hydrogen supply for high- temperature processes and combined heat- power generation
	3.4. Electric power sector: flexible demand balancing through hydrogen storage	FI [2.2→3.4]	Supports grid flexibility through large-scale storage and power-to-gas integration
2.3. Integration with renewable energy sources for green hydrogen production	3.2. Commercial and public sector: hospitals, schools, municipal buildings	FI [2.3→3.2]	Ensures decarbonization impact and climate policy compliance in public-sector heating applications
2.4. Deployment of pilot heating generation facilities with H _2_-ready cogeneration and heat pumps	3.1. Residential sector: hydrogen heating in new constructions and retrofitted households	FI [2.4→3.1]	Demonstrates technical and economic feasibility of H _2_-heating in household-scale applications
3.1. Residential sector: hydrogen heating in new constructions and retrofitted households	4.1. Public awareness campaigns and stakeholder engagement	FI [3.1→4.1]	Requires strong communication strategies to ensure household-level acceptance and reduce public skepticism
	4.2. Workforce training in hydrogen heating installation, operation, and safety	FI [3.1→4.2]	Necessitates the development of skilled labor to ensure proper installation, maintenance, and end-user support
3.2. Commercial and public sector: hospitals, schools, municipal buildings	4.3. Public-private partnerships (PPPs): integration of business, science, and government	FI [3.2→4.3]	Benefits from structured PPPs to co-finance projects, support institutional learning, and scale solutions across public assets
3.3. Industry: process heat and synergy with hydrogen use in manufacturing	4.2. Workforce training in hydrogen heating installation, operation, and safety	FI [3.3→4.2]	Demands highly specialized operational skills to manage hydrogen safely in industrial heat applications
3.4. Electric power sector: flexible demand balancing through hydrogen storage	4.3. Public-private partnerships (PPPs): integration of business, science, and government	FI [3.4→4.3]	Relies on collaborative innovation ecosystems to integrate hydrogen storage into grid-level balancing strategies

* Developed by the authors based on Figure 3.

One such case of how governmental backing can hasten such transitions is the H2HEAT project, funded by regional policy initiatives in the Canary Islands. In this instance, the application of hydrogen technologies was driven by policy-mandated requirements to transition away from diesel-based systems. Overall, despite substantial challenges that necessitate multi-disciplinary solutions, hydrogen heating is a feasible route to the decarbonization of the heating sector. Stakeholders can be best served by hydrogen's future potential as a critical element in heating solutions if they solve such issues through strategic proposals.

## Conclusion

Hydrogen heat systems are also advancing rapidly and possess promising solutions to decarbonize residential, commercial, and industrial heating. Hydrogen heating technology's current status, comprising green hydrogen manufacture using electrolysing, linkage to existing installed gas networks, combined heat and power (CHP) systems, and heat pumps' potential contribution, has been debated in the above context. Despite significant advances, major technical and economic barriers persist, including the expense of electrolyzers and renewable electricity, limitations of current infrastructure, compatibility of materials, and requests for standardized safety regimes.

Relative assessment of hydrogen with respect to other low-carbon pathways, such as direct electrification and methanol-based solutions, highlights the importance of situational deployment strategies. Hydrogen is not always the most efficient or least costly solution in all cases, but it has distinct advantages in hard-to-electrify areas and for seasonal storage in colder regions.

An alternative to direct hydrogen combustion in boilers is the use of electric heaters powered by electricity generated via Proton Exchange Membrane (PEM) fuel cells, which convert hydrogen and air into electricity with only water as a byproduct. This approach offers several advantages, particularly in terms of higher overall system efficiency and zero local emissions. PEM fuel cells typically operate at 40–60% electrical efficiency and can be combined with heat recovery systems to improve total energy utilization ( Asghar *et al.*, 2025; Singla *et al.*, 2021). Using the generated electricity to power high-efficiency electric heaters or heat pumps provides flexible and precise thermal management, especially in building environments where emissions, noise, and space constraints are critical considerations.

However, this pathway introduces additional system complexity and capital cost due to the need for fuel cells, power conditioning, and thermal-electric integration. Furthermore, fuel cell durability and hydrogen purity requirements present operational challenges. Thus, while PEM-based electric heating is a compelling low-emission alternative for certain applications—such as off-grid residential or sensitive urban areas—it may not always be the most cost-effective or scalable solution for large-scale or high-temperature industrial heating, where direct combustion still provides simplicity and high thermal output.

Hydrogen fuel cells, particularly Proton Exchange Membrane (PEM) and Solid Oxide Fuel Cells (SOFCs), are increasingly viewed as a next-generation solution for both stationary and mobile energy applications due to their high efficiency and near-zero emissions. Unlike hydrogen combustion in boilers—which involves high-temperature oxidation and the potential formation of nitrogen oxides (NOₓ)—fuel cells electrochemically convert hydrogen and oxygen into electricity and water, with no combustion-related pollutants. This makes them especially attractive in urban and environmentally sensitive zones ( Asghar *et al.*, 2025; Singla *et al.*, 2021; Yadav *et al.*, 2022).

In terms of efficiency, hydrogen boilers typically achieve 85–95% thermal efficiency, while PEM fuel cells can reach 40–60% electrical efficiency and, when combined in cogeneration systems, can exceed 85% total efficiency by utilizing waste heat. However, fuel cells operate best under stable load conditions and require high-purity hydrogen, making them more complex and expensive to implement at scale compared to the relatively simple and robust hydrogen boiler systems.

Ultimately, while fuel cells offer superior environmental performance and modularity, their deployment in heating applications may be best suited to low-emission zones, distributed energy systems, or integrated smart grids. In contrast, hydrogen combustion in CHP units or hybrid boiler-heat pump systems remains more practical in high-demand or industrial contexts where infrastructure and operational simplicity are prioritized.

Strategic policy backing is critical to facilitate investment, rationalize regulation convergence, and stimulate market deployment. Also critical is the role of public-private partnerships to stimulate innovation, derisk infrastructure upgrading, and establish public trust. Global cooperation can propel by facilitating collective learning, technology scaling, and coordinated funding mechanisms.

In this broader context, the Canary Islands' H2HEAT project is a shining and timely example of hydrogen heating at scale with robust government backing. The project shows how green hydrogen, generated from renewable sources and used in a hospital setting, can achieve carbon reduction goals as well as required infrastructure needs. Through combining cutting-edge electrolysis, CHP integration, and local stakeholders, H2HEAT demonstrates how local activities can be aligned with global decarbonization.

Policymakers should focus on creating clear, long-term policies that integrate hydrogen into energy strategies, providing financial incentives like subsidies to support early projects and harmonizing safety regulations to facilitate large-scale adoption. Industry stakeholders must invest in R&D to reduce hydrogen production and storage costs, retrofitting existing infrastructure for hydrogen compatibility, and collaborating with renewable energy providers for cost-efficient hydrogen production. Researchers should concentrate on developing cost-effective production methods, exploring regional economic variations, assessing the life-cycle environmental impacts of hydrogen, and addressing safety and material challenges to ensure the scalability and sustainability of hydrogen heating systems.

Ultimately, widespread hydrogen-based heating will only be realized through a unified, multi-sectoral effort by policymakers, industry leaders, researchers, and communities. It is only with such collaborative effort that hydrogen can realize its full potential as a cornerstone of a sustainable and low-carbon energy future.

This review describes that hydrogen heating is at a crossroads of technological change, energy policy, and socio-economic transformation. Its role in the decarbonization of heating is not marginal or ubiquitous but highly dependent on local infrastructure, market preparedness, and long-term climate policies. The path ahead must include a system-level approach that balances technical feasibility with economic viability, regulatory consistency, and social acceptance. Continuing interdisciplinary research, cross-border collaboration, and adaptive policy frameworks will be essential to position hydrogen as an scalable, reliable, and equitable choice in the future energy industry.

## Ethics and consent

Ethical approval and consent were not required

## Data Availability

No data associated with this article.
