# Supplementary material for: Hydrogen for Heating: Technologies, Challenges, and Opportunities
Source: Open Res Eur. 2026 Jan 15;5:143. Originally published 2025 May 28. [Version 3] doi: 10.12688/openreseurope.20432.3 (PMC12715400; doi:10.12688/openreseurope.20432.3)
Supplement: Peer review history

# Reviewer report: Reviewer response for version 3

**DOI:** 10.21956/openreseurope.24563.r68142

**Published:** 2026-01-20

**Authors:** Daogui Tang, Weiyi Cui

**Affiliations:** 1 Wuhan University of Technology, Wuhan, Hubei, China; 2 School of Transportation and Logistics Engineering, Wuhan University of Technology (Ringgold ID: 12565), Wuhan, Hubei, China

Thank you very much for your response and revisions. Manuscript was suitable to proceed further.

Is the review written in accessible language?

Yes

Are all factual statements correct and adequately supported by citations?

Partly

Are the conclusions drawn appropriate in the context of the current research literature?

Yes

Is the topic of the review discussed comprehensively in the context of the current literature?

Yes

Reviewer Expertise:

hydrogen energy, integrated multi-energy systems

We confirm that we have read this submission and believe that we have an appropriate level of expertise to confirm that it is of an acceptable scientific standard.

# Reviewer report: Reviewer response for version 2

**DOI:** 10.21956/openreseurope.22433.r65931

**Published:** 2026-01-07

**Authors:** Daogui Tang, Weiyi Cui

**Affiliations:** 1 Wuhan University of Technology, Wuhan, Hubei, China; 2 School of Transportation and Logistics Engineering, Wuhan University of Technology (Ringgold ID: 12565), Wuhan, Hubei, China

The topic of this manuscript is timely and practically relevant. It covers three key dimensions: technology, economics, and policy/regulation, and attempts to use the H2Heat project as a case study to connect technical discussion with policy implications. However, in its current form, the manuscript reads more like a compiled narrative summary of policy documents and selected literature rather than a rigorous review article. The transparency of the review methodology is insufficient, and there are multiple issues related to language quality and terminology consistency, which collectively weaken the scientific rigor and readability. Overall, I recommend indexing after revision.

 (1) The “Materials and methods” section is largely a descriptive sequence of cited studies, but it does not provide essential information on how the literature and evidence were collected and filtered (e.g., databases consulted, time span, search keywords, inclusion/exclusion criteria, and the number and types of sources). It is recommended to provide a “Summary Scope and Evidence Sources Table” to prevent readers from perceiving the conclusions as derived from “selective citation".

 (2) The manuscript contains several abrupt heading combinations and paragraph transitions that increase the reading burden. For example, in the “CHP and heat pump” section, the text first introduces “Other technologies,” which is structurally confusing. In addition, “technological advances” are reiterated multiple times in later sections with limited incremental content. The authors are encouraged to reorganize the manuscript around a clearer main storyline and align section headings with the actual content.

 (3) Some numerical claims are presented without specifying the assumptions or boundary conditions under which they hold, which reduces credibility. For instance, the manuscript states: “If pure hydrogen replaces natural gas in pipelines, the volumetric leak-age rate (m3/s) would increase by approximately 2.8 times. However, the associated energy loss would decrease by around 9.2%, reflecting the lower specific energy losses during hydrogen transport compared to methane” The authors should clarify under what conditions these factors (2.8 times and 9.2%) were derived.

 (4) There are multiple spelling and formatting problems (e.g., “A breaf…,” “socalled…,” and unnecessary conversational phrasing), as well as inconsistent notation such as H2/H _2_, CH4/CH _4_ and occasional improper hyphenation/line breaks. A full consistency check and professional language editing are recommended. Moreover, the meaning of “hydroxy” is unclear and appears to be technically inappropriate in the following sentence: “The assessment of technological advances in the use of hydrogen, referred to as **hydroxy** , for heating purposes involves a comprehensive evaluation of recent innovations and persistent challenges.” The authors should clarify what is meant by “hydroxy” (or correct it if it is a mistake) and ensure that terminology is accurate throughout.

 (5)The conclusion mentions comparisons with alternative decarbonization pathways (e.g., electrification and methanol), but a clear comparative framework is missing. The authors are encouraged to add explicit comparison dimensions (e.g., delivered heat cost, lifecycle emissions, retrofit complexity, infrastructure readiness, safety/regulatory burden) and include a summary comparison table in the main text.

Is the review written in accessible language?

Yes

Are all factual statements correct and adequately supported by citations?

Partly

Are the conclusions drawn appropriate in the context of the current research literature?

Yes

Is the topic of the review discussed comprehensively in the context of the current literature?

Yes

Reviewer Expertise:

hydrogen energy, integrated multi-energy systems

We confirm that we have read this submission and believe that we have an appropriate level of expertise to confirm that it is of an acceptable scientific standard, however we have significant reservations, as outlined above.

# Response

**Published:** 2026-01-08

**Authors:** Oleh Sokil

Dear Dr. Tang and Dr. Cui, Thank you for your valuable feedback on our manuscript. We appreciate your time and effort in reviewing our work and for your insightful suggestions. We have made several revisions based on your recommendations, particularly in improving the **Materials and Methods** section by clarifying the literature search process and providing a more transparent summary of evidence sources. Additionally, we reorganized sections for better clarity and consistency, addressing the abrupt transitions and redundant discussions you pointed out. We have also ensured that numerical claims are now more clearly explained, including the conditions under which they apply, and have corrected any language inconsistencies or technical inaccuracies, such as the "hydroxy" terminology. Finally, we have added a comparative framework to the conclusion, including a summary table comparing hydrogen heating with alternative decarbonization pathways. Thank you once again for your constructive feedback. We hope the revisions have addressed your concerns and that the manuscript is now stronger. Sincerely, Oleh Sokil

# Reviewer report: Reviewer response for version 2

**DOI:** 10.21956/openreseurope.22433.r65925

**Published:** 2026-01-07

**Authors:** Maoqiang Jiang

**Affiliations:** 1,Wuhan University of Technology, Wuhan, Hubei, China

This manuscript provides a timely and comprehensive review of hydrogen's role in decarbonizing the heating sector. It successfully covers a wide range of critical aspects, including production pathways (with a focus on green hydrogen), storage and distribution technologies, end-use applications (CHP, boilers, heat pumps), and the associated economic, regulatory, and safety challenges. The integration of a concrete case study (the H2Heat project) strengthens the practical relevance of the discussion. 

 However, to meet the standards of a rigorous academic review article, the manuscript requires significant revisions to enhance its scholarly contribution, analytical depth, and structural clarity. 

 Comments and Suggestions

 1. The current organization leads to considerable redundancy, with similar discussions on electrolysis efficiency, infrastructure challenges, and policy contexts reappearing across the Introduction, Results, and technological sections. Suggestion: Radically restructure the manuscript. Consider clear, dedicated sections such as: 1. Technological Landscape (production, storage, end-use); 2. Integrated Challenges (technical, economic, safety, regulatory); 3. Opportunities, Case Studies & Pathways Forward (deep dive into H2Heat, comparative analysis of deployment scenarios). This will create a more logical and compelling narrative flow.

 2. Many key statements, especially quantitative claims, lack direct and appropriate citations, undermining academic credibility. Suggestion: Systematically audit the text for assertions related to costs, efficiencies, performance data (e.g., leak rates, tank specifications, electrolyzer efficiency ranges, retrofit costs) and provide citations from authoritative sources (peer-reviewed literature, IEA/IRENA reports, official strategies).

 3. The analysis of various technologies (PEM vs. Alkaline electrolysis, Type I-V tanks, boilers vs. fuel cells vs. CHP) is fragmented. Suggestion: Introduce synthesizing tables or summary figures that directly compare key parameters: Technology Readiness Level (TRL), efficiency range (electrical/thermal), cost drivers (CAPEX/OPEX), advantages, limitations, and ideal use cases. This is crucial for reader comprehension and fulfills a core purpose of a review.

 4. While cost is flagged as a barrier, the analysis remains qualitative. Suggestion: Enhance the economic discussion by incorporating representative quantitative data ranges (e.g., current and projected LCOH in €/kg, electrolyzer CAPEX in €/kW, estimated grid retrofit costs). Briefly discuss drivers for cost reduction (scale, learning curves, electricity prices).

 5. The article is text-heavy, lacking visual summaries that could greatly improve readability and comprehension. 

 6. The "Materials and methods" section does not describe a reproducible review methodology. Suggestion: Rewrite this section to detail the systematic approach: literature search strategy (databases, keywords, date ranges), inclusion/exclusion criteria for selecting studies, and the analytical framework used to categorize and evaluate the information (e.g., PESTEL analysis, multi-criteria assessment).

 The manuscript addresses a topic of high importance and contains valuable information. However, in its current form, the issues outlined above—particularly regarding organizational coherence, methodological transparency, and analytical synthesis—are substantial. I therefore recommend "Approved with Reservations".

Is the review written in accessible language?

Yes

Are all factual statements correct and adequately supported by citations?

Yes

Are the conclusions drawn appropriate in the context of the current research literature?

Yes

Is the topic of the review discussed comprehensively in the context of the current literature?

Yes

Reviewer Expertise:

Onboard hydrogen utilization and safty.

I confirm that I have read this submission and believe that I have an appropriate level of expertise to confirm that it is of an acceptable scientific standard, however I have significant reservations, as outlined above.

# Response

**Published:** 2026-01-08

**Authors:** Oleh Sokil

Dear Dr. Jiang, Thank you very much for your detailed and constructive feedback on our manuscript. We truly appreciate the time and effort you dedicated to reviewing our work, as well as your insightful suggestions. Your comments have been incredibly helpful in identifying areas for improvement, and we have carefully considered each point you raised. We have made several changes to the manuscript based on your recommendations. Specifically, we have restructured the manuscript to improve its flow and coherence, addressing redundancies and consolidating the discussions on electrolysis efficiency, infrastructure challenges, and policy context into more focused sections. We have also worked to strengthen the citation support for many of the key quantitative claims and have added references from authoritative sources such as IEA and IRENA. While we have implemented significant revisions, some aspects, particularly in terms of deeper analysis and the inclusion of synthesizing tables or figures, are areas we plan to address further in subsequent studies and articles. We also acknowledge the need for more detailed economic data, which will be incorporated in future work as we continue to refine our analysis. Again, thank you for your valuable feedback. We believe the manuscript is now stronger as a result, and we hope it will meet your expectations. Sincerely, Oleh Sokil

# Reviewer report: Reviewer response for version 2

**DOI:** 10.21956/openreseurope.22433.r64174

**Published:** 2025-12-18

**Authors:** PEEYUSH PHOGAT

**Affiliations:** 1 Netaji Subhas University of Technology, New Delhi, Delhi, India

**Summary of the Article**

 The manuscript provides a broad and detailed overview of hydrogen-based heating technologies and their role in global decarbonization strategies. It reviews:

- Hydrogen production pathways (green, blue, etc.)
- Storage technologies including compressed gas tanks, LOHCs, metal hydrides
- Integration into existing natural gas networks, retrofitting challenges, and safety issues
- Technological advances in CHP, fuel cells, and hybrid CHP–heat pump systems
- Regulatory frameworks and policy developments across Spain, EU, and global contexts
- Economic feasibility, infrastructure requirements, and lifecycle emissions considerations
- A detailed case study of the EU-funded H2HEAT project

The review concludes that hydrogen heating can be viable in specific contexts—particularly industrial heat, seasonal storage, and regions with abundant renewables—while emphasizing the need for harmonized regulation, investment, and public–private collaboration.

**Major Comments**

**1. Improve structural organization and reduce repetition**

 Sections such as “CHP and heat pump,” “Technological advancements,” and parts of the introduction reiterate similar content on electrolysis, infrastructure challenges, and policy context. Consolidating overlapping paragraphs would significantly improve clarity and flow.

**2. Strengthen citation support for quantitative claims**

 Several statements require stronger or more precise referencing, including:

- Hydrogen leak rate comparisons and energy loss percentages
- Electrolysis efficiencies and cost trends
- Thermal and electrical efficiencies of CHP and PEM fuel cells
- Noise levels from electrolysis and CHP units
  Explicitly citing primary literature or authoritative reports (IEA, EU Hydrogen Strategy, DOE) would increase robustness.

**3. Clarify distinctions between technologies**

 The manuscript sometimes blends discussion of hydrogen boilers, CHP units, PEM fuel cells, and heat pumps without clearly distinguishing their operating conditions, use cases, and performance boundaries. Authors should:

- Present a comparative table of key parameters (efficiency, cost drivers, temperatures, readiness levels).
- Clearly delineate which technologies are short-term deployable versus long-term prospective.

**4. Add critical depth to economic feasibility analysis**

 Although economic challenges are noted, the analysis would benefit from:

- Numerical cost comparisons (€/kg H₂, CAPEX of electrolyzers, cost of retrofitting gas grids)
- Regional cost variability tied to renewable electricity prices
- Clearer differentiation between centralized vs. decentralized hydrogen production economics

**5. Improve figures and ensure consistency with text**

 Figures 1–3 are useful but require:

- Clearer captions explaining their significance
- Consistent units and color schemes
- Higher resolution for enhanced legibility (some improvements were made but further refinement is needed)

**6. Enhance clarity in the Conclusions section**

 The conclusions are comprehensive but could be more concise. Consider organizing into bullet-pointed recommendations for:

- Policymakers
- Industry stakeholders
- Researchers
  This will allow readers to better extract actionable insights.

Is the review written in accessible language?

Partly

Are all factual statements correct and adequately supported by citations?

Partly

Are the conclusions drawn appropriate in the context of the current research literature?

Yes

Is the topic of the review discussed comprehensively in the context of the current literature?

Yes

Reviewer Expertise:

Electrochemistry, Material science, HER and OER

I confirm that I have read this submission and believe that I have an appropriate level of expertise to confirm that it is of an acceptable scientific standard, however I have significant reservations, as outlined above.

# Response

**Published:** 2026-01-08

**Authors:** Oleh Sokil

Dear Dr. Phogat, Thank you for your thoughtful and constructive feedback on our manuscript. We greatly appreciate your time and effort in reviewing our work. We have carefully considered your comments and made several revisions to improve the clarity, structure, and depth of the article. We worked on improving the structural organization of the manuscript, consolidating overlapping content to enhance the flow and readability. Additionally, we have strengthened the citation support for certain claims and clarified distinctions between the technologies discussed, ensuring that the different systems are presented with more clarity regarding their use cases and performance. We also expanded the economic analysis to provide a clearer view of the cost aspects, including regional variations and key economic drivers. In response to your suggestion about the figures, we have made adjustments to improve their clarity and consistency with the text, including refining captions and ensuring consistency in units and visual elements. Finally, we restructured the conclusion to provide more concise recommendations for policymakers, industry stakeholders, and researchers, making it easier for readers to extract actionable insights. Thank you once again for your valuable feedback. We hope the revised manuscript now better addresses your concerns. Sincerely, Oleh Sokil

# Reviewer report: Reviewer response for version 2

**DOI:** 10.21956/openreseurope.22433.r56841

**Published:** 2025-08-26

**Authors:** Patrick Ehi Imoisili

**Affiliations:** 1 University of Johannesburg, Auckland Park, Gauteng, South Africa

The article presents a timely and insightful exploration of hydrogen’s potential in decarbonizing heating across residential, commercial, and industrial sectors. It offers a comprehensive overview of current technologies, economic considerations, and regulatory landscapes, supported by relevant case studies such as the H2Heat project. The integration of green hydrogen into systems like CHP and heat pumps is well-articulated, and the discussion acknowledges both progress and persisting challenges. Overall, the article is well-researched, clearly written, and makes a valuable contribution to the discourse on sustainable energy transitions. I recommend it for indexing with only minor revisions.

 1) Add recent research in Hydrogen

Is the review written in accessible language?

Yes

Are all factual statements correct and adequately supported by citations?

Yes

Are the conclusions drawn appropriate in the context of the current research literature?

Yes

Is the topic of the review discussed comprehensively in the context of the current literature?

Yes

Reviewer Expertise:

Sustainable Energy, Green Hydrogen

I confirm that I have read this submission and believe that I have an appropriate level of expertise to confirm that it is of an acceptable scientific standard.

## References

[1] : South Africa clean energy transition: The future of green hydrogen energy technology. *Energy Reports* .2025;13: 10.1016/j.egyr.2025.05.005 5501-5511 10.1016/j.egyr.2025.05.005

[2] : Renewable Energy Technologies: Opportunities and Challenges for South Africa. *(ICSEMD)* . https://doi.org/10.4028/p-rulYG5 10.4028/p-rulYG5

# Response

**Published:** 2025-08-26

**Authors:** Oleh Sokil

Dear Dr. Patrick Ehi Imoisili, On behalf of our research team, I would like to sincerely thank you for your thoughtful and positive review of our article on hydrogen. We deeply appreciate the time and effort you devoted to evaluating our work, as well as your encouraging comments. Your feedback not only strengthens the quality of our publication but also motivates us to continue advancing our research in this important field. We are truly grateful for your support and professional contribution.   With kind regards, Oleh Sokil

# Reviewer report: Reviewer response for version 1

**DOI:** 10.21956/openreseurope.22110.r54837

**Published:** 2025-06-12

**Authors:** Amandeep S Oberoi

**Affiliations:** 1 Thapar Institute of Engineering and Technology, Patiala, Punjab, India

The presented manuscript is focused on unwinding the role of hydrogen in decarbonize heating and delve into literature pertinent to hydrogen production, storage and distribution. As such the topic is worth exploring and gives a comprehensive reporting on advancements in infrastructure prepared for hydrogen, electrolyzer efficiency, and renewable energy-based hybrid energy systems. I found most of the paper to be well written however, it requires a revision before recommending it for indexing. I urge authors to revise the paper as per the below comments:

 1) Figure-1, Label on the y-axis is missing. Is the temperature marked in degree Celsius or K or F? 

 2) Text in Figure-2 and 3 is not readable. Kindly increase the size and resolution of these images.

 3) Energy expenditure associated with compressing the hydrogen gas or storing it in liquid state at cryogenic temperatures is not talked about. Comparatively, solid stage hydrogen adsorption require lesser or no energy expenditure. Kindly throw some light on it.

 4) Cost of electricity produced from renewable sources for powering electrolyzer may vary geographically. For an instance, cost of electricity produced from solar PV in countries (like, India) experiencing more sunlight would be low compared to the countries near north or south pole (for example, Canada). So, would be the variation in price of Green Hydrogen production. Kindly comment on it and add the relevant literature in the paper.

 5) Cost of different types of compressed hydrogen storage tanks also varies as per the pressure they can handle. For example, Type-I, Type-II, Type-III, Type-IV, and Type-V tanks used for compressed H2 storage. Kindly elaborate on it and include it in the paper.

 6) Adding a polymer lining by thermal spray coating or similar methods could reduce the problem of hydrogen embrittlement in the existing metallic pipelines used for CNG transportation. Think on these lines and added literature on it in the paper.

 7) Instead of combusting hydrogen in boilers, why can't electric heaters be used for heating powered by electricity produced using PEM fuel cells that required H2 and air? Comment on it please.

 8) Hydrogen fuel and fuel cell technology are foreseen as future to replace conventional fossil fuels for stationary and mobile applications. In particular, fuel cell could produce electricity using H2 and air by giving out just water and absolutely no emissions. Kindly compare it with the firing of hydrogen in boilers for heating and add the supporting lietrature.

Is the review written in accessible language?

Yes

Are all factual statements correct and adequately supported by citations?

Partly

Are the conclusions drawn appropriate in the context of the current research literature?

Partly

Is the topic of the review discussed comprehensively in the context of the current literature?

Yes

Reviewer Expertise:

electrochemical hydrogen adsorption in porous mediums, green hydrogen production, PEM electrolyser, PEM fuel cell, reversible fuel cell.

I confirm that I have read this submission and believe that I have an appropriate level of expertise to confirm that it is of an acceptable scientific standard, however I have significant reservations, as outlined above.

## References

[1] : Hydrogen fuel and fuel cell technology for cleaner future: a review. *Environmental Science and Pollution Research* .2021;28(13) : 10.1007/s11356-020-12231-8 15607-15626 10.1007/s11356-020-12231-8 33538968

[2] : Electrochemical hydrogen storage: Achievements, emerging trends, and perspectives. *International Journal of Energy Research* .2022;46(12) : 10.1002/er.8407 16316-16335 10.1002/er.8407

[3] : Cost–benefit comparison of fuel cell–based and battery‐based renewable energy systems. *International Journal of Energy Research* .2022;46(2) : 10.1002/er.7291 1736-1755 10.1002/er.7291

[4] : A Review on the Cost Analysis of Hydrogen Gas Storage Tanks for Fuel Cell Vehicles. *Energies* .2023;16(13) : 10.3390/en16135233 10.3390/en16135233

[5] : A Comprehensive Literature Review on Hydrogen Tanks: Storage, Safety, and Structural Integrity. *Applied Sciences* .2024;14(20) : 10.3390/app14209348 10.3390/app14209348

[6] : A review of metallic tanks for H2 storage with a view to application in future green shipping. *International Journal of Hydrogen Energy* .2021;46(9) : 10.1016/j.ijhydene.2020.11.168 6151-6179 10.1016/j.ijhydene.2020.11.168

[7] : Review on large-scale hydrogen storage systems for better sustainability. *International Journal of Hydrogen Energy* .2023;48(85) : 10.1016/j.ijhydene.2023.04.304 33223-33259 10.1016/j.ijhydene.2023.04.304

# Response

**Published:** 2025-06-19

**Authors:** Oleh Sokil

Ось коротка відповідь рецензенту англійською мовою:

**Response to Reviewer** We sincerely thank Dr. Amandeep S. Oberoi for the careful review and constructive comments provided on our manuscript. We highly appreciate the thoughtful suggestions, which have contributed significantly to improving the clarity and completeness of our work. All of the reviewer’s recommendations have been carefully addressed and incorporated into the final version of the manuscript. This includes textual additions, updated figures with improved resolution, and integration of relevant literature to reflect the points raised. We are grateful for the insights and believe that the revised manuscript is now much stronger as a result. Thank you once again for your valuable feedback and support.
